# Chemometric Analysis of Fatty Acid Composition of Raw Chicken, Beef, and Pork Meat with Plant Extract Addition during Refrigerated Storage

**DOI:** 10.3390/molecules26164952

**Published:** 2021-08-16

**Authors:** Małgorzata Muzolf-Panek, Anna Kaczmarek

**Affiliations:** Department of Food Quality and Safety Management, Faculty of Food Science and Nutrition, Poznań University of Life Sciences, Wojska Polskiego 31, 60-637 Poznań, Poland; anna.kaczmarek@up.poznan.pl

**Keywords:** fatty acids, chicken, pork, beef, plant extracts, time effect, principal component analysis

## Abstract

During the shelf-life, meat undergoes a number of processes that negatively affect the quality of the product, including fatty acid composition. The application of various plant extracts in meat could affect the changes of fatty acids during storage. Thus, the aim of this study was to investigate the effect of various spice and herb extracts on fatty acid composition in raw pork, beef, and chicken meat when stored at 4 °C for 13 days. Based on multivariate statistical analysis, two datasets were extracted from each type of meat. One dataset included samples with allspice, bay leaf, black seed, cardamom, caraway, clove, and nutmeg with the high share of total MUFA (monounsaturated fatty acids) in chicken and pork meat and high MUFA and PUFA (polyunsaturated fatty acids) contribution in beef meat after storage. The second dataset included basil, garlic, onion, oregano, rosemary, and thyme with high PUFA share in chicken and pork meat and high SFA (saturated fatty acids) contribution in beef meat. From the regression analysis, a significant effect of time on fatty acid composition in meat was reported. Generally, the rates of fatty acid changes were dependent on the plant extract incorporated into the meat. The most visible effect of plant extracts was obtained in chicken meat. In chicken meat with plant extracts, the rates of SFA and PUFA changes with time were slower compared to the control sample. In summary, the fatty acid composition of intramuscular fat varied during storage, and the addition of plant extracts significantly affected the rate of these changes, which was dependent on the meat matrix.

## 1. Introduction

Global consumption of meat has been continuously on the upward trend, although some percentage of the human population chooses not to consume meat (vegan, vegetarian, or even flexitarian lifestyle has increased), and consumers’ concerns about the animal treatment and the impact of meat production on the environment have gained more attention, especially in developed countries [1]. Additionally, other factors such as rise in the consumers’ knowledge about the effect of diet on human health also have some effects on the global meat market.

Meat is a very complex matrix, and the health consequences of meat consumption are multidimensional. It is rich in proteins containing essential amino acids and fats with a broad range of fatty acid compositions [2]. Thus, on the one hand, meat is a good source of energy, unsaturated fatty acids (UFA), proteins of high nutritional quality, heme iron, zinc, and vitamins, but on the other hand, meat fat contains saturated fatty acids (SFA), which could influence negatively human health [3]. In recent years, red meat consumption has been associated with a higher risk of obesity, coronary heart diseases, and cancer, although it should be emphasized that the results of epidemiological studies are not always consistent [4]. However, fat and fatty acids are involved in many vital processes in the human body; for example, linoleic acid showed a negative dose-dependent relationship between the intake and the level of LDL fraction of cholesterol in humans [2,5]. The specific guidelines for fat and fatty acids composition were reviewed by the EFSA (European Food Safety Authority) [5]. The authority has recommended reduction in SFA and trans fatty acid intakes and proposed adequate intakes of linoleic and linolenic acids indispensable for our proper growth and development [5].

Fatty acid composition varied among animal species and breed [6], type of muscle and cut of meat [7,8], animal diet [9,10], fat content [6,11], and sample handling [8], especially thermal treatment [7]. The most popular meat types worldwide are chicken, pork, and beef [1]. The fat content in these types of meat (unprocessed) varies in the broad range from 2.62 g to 22.07 g/100 g for chicken breast and beef ribeye, respectively [3]. Among the three types of meat, the highest share of SFA was observed for beef, and the SFA share in total fatty acids was accounted for about 46–47% [7,12]. In pork, the percentage of SFA equaled 40–43% [6,8,13], and the lowest level of SFA was assessed in chicken meat, especially breast, with the share of 27–30% [14,15]. The highest share of UFA, especially PUFA (polyunsaturated fatty acids), was demonstrated in chicken meat (56–61% of total fatty acids, including 26–30% of PUFA) [14,15], which makes poultry highly favorable from the nutritional point of view [2]. Beef and pork possess a significantly lower percentage of UFA (especially PUFA) and lower value of PUFA/UFA index than chicken meat. However, in both meat types, the MUFA (monounsaturated fatty acids) share is relatively high and equals 39–48% for beef [7,12] and 39–52% for pork [8,11,13], depending on the meat cut.

Although the high content of PUFA is beneficial for human health, it makes the meat highly susceptible to the oxidation process, which negatively affects the quality of the product and, finally, consumers’ health. The exposure to oxygen or heat and the degree of fatty acid unsaturation affects the rate of lipid oxidative stability and could result in the change in FA composition by the decline in UFA content and an increase in SFA level. As was pointed out by Dominguez et al. [16] in their review, the susceptibility of meat to the oxidation process resulted more from the unsaturation of fatty acids than the overall fat content in meat. Thus, the challenge of the food industry is to maintain meat quality in relation to UFA stability during shelf-life, particularly in aerobic conditions. Various technological practices were applied to this end, such as packaging in a modified atmosphere or under a vacuum [14,17]. However, literature data gave inconsistent results. Chmiel et al. [14] reported no relationship between the changes of the fatty acid profile in chicken meat packed using the aforementioned methods, whereas Orkusz et al. [17] found vacuum packaging to be an effective method to maintain the constant fatty acid composition in goose meat during storage. Another way for sustaining the quality of meat during the storage period is the application of antioxidants to the product. This could be performed in two modes. The first one, indirect mode, is the incorporation of antioxidants into the animals’ diet. The effectiveness of the method depends on the absorption, distribution, biotransformation, and excretion of the compounds by the animal body. The second one is the direct mode, the addition of antioxidants to the food product, which is still the most popular way of food fortification with the active antioxidant compounds among the food producers. In the European Union, the antioxidants legally permitted in minced meat are ascorbic acid and its salts (identified by E300, E301, and E302) and citric acid and its salts (identified by the E330, E331, E332, and E333) [18]. All compounds are chemically synthesized. The antioxidants approved for use in meat and meat products in the USA are butylated hydroxyanisole (BHA), butylated hydroxytoluene (BHT), propyl gallate (PG), tert-butylhydroquinone (TBHQ), and tocopherols (only in poultry) [19]. As consumers are growing in their distrust for using chemically synthesized compounds in food, the “natural” and “clean label” claims for food have gained consumers’ increasing interest [19,20,21]. This led to a search for natural alternatives, and considerable research on the application of various plant extracts in meat is needed.

Thus, the aim of this study was to investigate the fatty acid composition of raw meat samples: chicken, pork, and beef with the addition of extracts prepared from 13 commonly used spices and herbs: allspice, basil, bay leaf, black seed, cardamom, caraway, cloves, garlic, nutmeg, onion oregano, rosemary, and thyme. The study was conducted during 13 days of storage at 4 °C. To put more insight into the data, PCA (principal component analysis) was performed as the first step of data analysis, which enabled visualization of results and detection of relationships between fatty acids and the sample (source of natural antioxidants). Moreover, the MLR (multiple linear regressions) models were obtained to show the effect of time on fatty acid changes in the samples.

## 2. Results

Fatty acid composition is an important index of the quality of meat. Its changes during storage could lead to the lowering of the nutritional quality of the product by the increase in SFA and decrease in UFA share. To counteract or slow down this negative trend, plant extracts as a source of antioxidants could be used. In this study, the effect of various spice and herb extracts on the fatty acid composition of raw chicken, pork, and beef meat were analyzed during refrigerated storage for 13 days.

**Figure 1:**
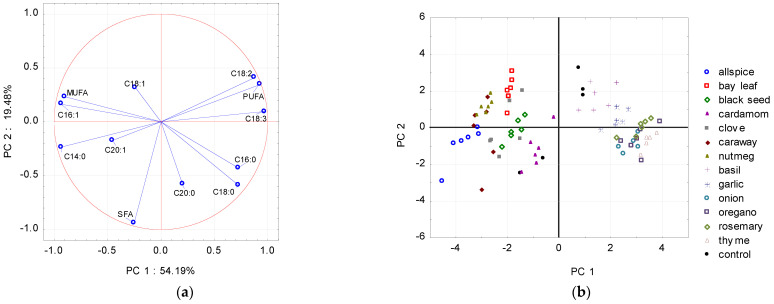
Projections of the (**a**) variables (fatty acids) and (**b**) scores (plant extracts) for raw chicken meat samples onto the factor plane defined by principal components (PC1 and PC2).

**Figure 2:**
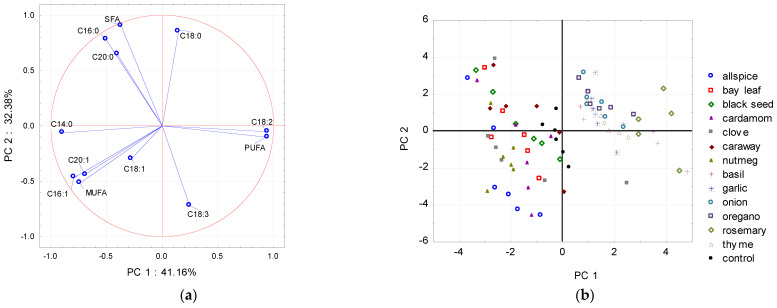
Projections of the (**a**) variables (fatty acids) and (**b**) scores (plant extracts) for raw pork meat samples onto the factor plane defined by principal components (PC1 and PC2).

**Figure 3:**
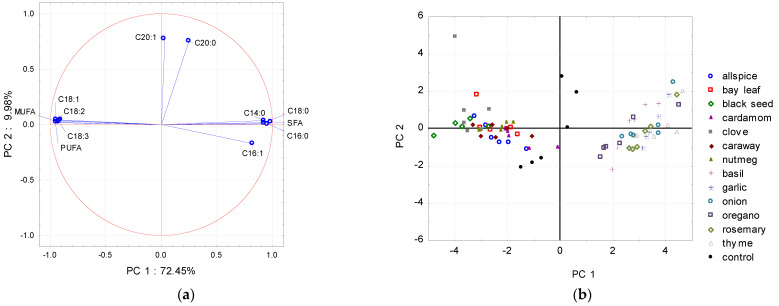
Projections of the (**a**) variables (fatty acids) and (**b**) scores (plant extracts) for raw beef meat samples onto the factor plane defined by principal components (PC1 and PC2).

To detect patterns between the extract added and the fatty acid composition (data from the whole storage period), PCA was applied. As shown in Figure 1, Figure 2 and Figure 3, PCA models revealed significant relationships between the fatty acid profiles and the type of the plant extract treatment. Using the graphical criterion, the first three principal components (PCs) were extracted for raw chicken and raw pork, and together they explained 86.5% and 84.5% of the total variance, respectively. For raw beef, the first two PCs had an eigenvalue greater than 1, and together they explained 82.5% of the total variance.

Based on the loaded values showing the correlation between fatty acid share and the corresponding factor coordinates (PCs), it could be stated that linoleic acid (C18:3), meristic acid (C14:0), palmitoleic acid (C16:1), total PUFA, and MUFA distributed raw chicken meat samples along to the PC1 (principal component 1) axis, whereas total SFA along to the PC2 (principal component 2) axis (Figure 1a).

Generally, for raw pork (Figure 2a), the projection of fatty acids onto the factor plane gave a similar pattern. The fatty acids of raw pork highly correlated with the PC1 were as followed: linoleic acid (C18:2), total PUFA, myristic acid (C14:0), palmitoleic acid (C16:1), and total MUFA, whereas total SFA, stearic acid (C18:0), and palmitic acid (C16:0) were highly correlated with the PC2.

According to the results presented in Figure 1b and Figure 2b, PC1 enabled the division of the chicken and pork meat samples dataset into two subsets. The first subset included samples treated with spices like allspice, bay leaf, black seed, clove, caraway, nutmeg, and cardamom (the latter sample only for raw pork meat) with the high level of MUFA in their total fatty acid profiles; and the second one included samples with garlic, onion, and herbs like basil, oregano, thyme, and rosemary with the high level of PUFA. The control samples for chicken and pork meat were placed close to the middle of the PC1 axis.

For raw beef, the projection of fatty acids along to the PC axes gave a different pattern (Figure 3a) than it was observed for chicken and pork samples. PC1 was highly correlated with total SFA, palmitic acid (C16:0), total PUFA, stearic acid (C18:0), oleic acid (C18:1), total MUFA, myristic acid (C14:0), linoleic acid (C18:2), linolenic acid (C18:3), and palmitoleic acid (C16:1), whereas arachidic (C20:0) and eicosenoic (C20:1) acids distributed samples along to the PC2 axis.

According to the PC1, the classification of the beef samples into the two subsets was visible (Figure 3b).

The first subset included beef samples with the high share of UFA (MUFA and PUFA), namely allspice, bay leaf, black seed, cardamom, clove, caraway, and nutmeg. The second subset included beef samples with a high share of SFA like C14:0, C16:0, and C18:0 in total fatty acid content, namely basil, oregano, rosemary, thyme, garlic, and onion. The control sample for beef was placed in the middle of the factor plane, showing moderate levels of all fatty acids.

The observations from PCA were confirmed by the results of general regression models (GRMs), which reported a significant effect of plant extract treatment (*p* ≤ 0.05) and time of storage (apart from C20:1 of raw chicken meat) on the fatty acid composition. All data are shown in Table 1, Table 2 and Table 3 as total mean values (calculated for the whole storage period). According to Table 1, the highest shares of PUFA were reported for the same subset of samples as shown in PCA and were in the range from 25.17% for onion to 25.78% for rosemary-treated chicken meat, which was significantly higher than the PUFA share of the control sample (23.29%). This could suggest the protective effect of basil, garlic, onion, oregano, rosemary, and thyme against the oxidation of PUFA in raw chicken meat. Although the levels of PUFA in allspice, bay leaf, black seed, clove, caraway, and nutmeg-treated samples were lower or not statistically different from the control sample, their absolute shares of MUFA in total fatty acid composition were higher than MUFA shares of control and other treated samples. This indicates that when allspice, bay leaf, black seed, clove, caraway, and nutmeg were added into the meat, the oxidation of PUFA occurred during storage to a greater extent than in control and other tested samples. The highest contribution among MUFA of raw chicken was of oleic acid (C18:1), from 38.2% for cardamom to 40.1% for bay leaf-treated chicken meat. Among PUFA, a considerable share of linoleic acid (C18:2) was observed, ranging from 18.4% (allspice) to 22.3% (rosemary). As for SFA, palmitic acid (C16:0) contributed the most to total SFA. Comparing to the control sample (30.4%), a significantly lower SFA share was observed for chicken meat with basil, bay leaf, and garlic (28.5%, 29%, and 29.5%, respectively), whereas significantly higher SFA contributions were reported after allspice and cardamom addition (31.6% and 31.5%, respectively) after storage.

Results of the fatty acid composition of raw pork samples shown in Table 2 also confirmed PCA observations as two datasets of pork samples stored at 4 °C could be determined based on MUFA and PUFA contribution in total fatty acids. Comparing to the control sample, basil-, oregano-, rosemary-, thyme-, garlic-, and onion-treated samples showed a high share of PUFA (9.9–12.3%) and a low level of MUFA (45.8–47.6%) during storage. For the control pork sample, PUFA accounted for 9.1% and MUFA for 49.0%. Thus, the same conclusions as for chicken meat samples could be achieved based on these observations as basil, garlic, onion, oregano, rosemary, and thyme slowed down the oxidation of PUFA in comparison to other samples. Similarly to chicken meat, the highest share of fatty acids in pork samples was ascribed to oleic acid (43.1–45.3%) among MUFA, linoleic acid (7.4–11.6%) among PUFA, and palmitic acid (24.5–26.1%) among SFA. The contribution of SFA and MUFA in total fatty acids of raw pork was significantly higher than in raw chicken meat, and the share of PUFA was more than two-fold lower.

Table 3 includes the fatty acid composition of raw beef samples. Total PUFA share of beef was the lowest among the three types of meat tested and accounted for from 2.8% to 5.5%. MUFA share in total fatty acids of beef samples was similar to the one reported for pork samples. SFA contribution in total fatty acids of beef ranged from 45.1% to 50.3% and was the highest among tested meat types, almost twice as high as for chicken meat. The most abundant fatty acids were linoleic acid (42.9–48.1%), palmitic acid (25–27.6%), and stearic acid (16.7–20.3%). The patterns detected by the aforementioned PCA could be confirmed by the data from Table 3. After storage of raw beef with allspice, bay leaf, black seed, cardamom, clove, caraway, and nutmeg, the decrease in UFA oxidation was observed. This was reflected by the higher shares of PUFA and MUFA and thus lower SFA contribution in total fatty acids in comparison to control and other tested samples. Finally, this suggested the beneficial effect of these seven treatments on the fatty acid composition of raw beef during storage.

Next, MLR analysis was performed to assess the effect of plant extracts on the rate of total SFA, MUFA, and PUFA changes during refrigerated storage. All results of regression analysis are shown in Table 4 and changes of the fatty acid composition of the samples with time in Supplementary Materials (Table S1). Based on the analysis of regression coefficients (β) for SFA, it could be noticed that in all meat samples, an increase in SFA shares with time reflected by the positive values of regression coefficients and a decrease in PUFA reflected by the negative values of regression coefficients occurred. The linear model was statistically significant (*p* ≤ 0.05) in almost all cases when analyzing SFA and PUFA data. Only for the cardamom sample in pork meat, the linear model for PUFA was not statistically significant (*p* = 0.25, R^2^ = 0.15) since the share of PUFA did not change during storage. When comparing the PUFA contribution of different control samples, it could be noticed that the loss of PUFA was faster in chicken meat (β = −0.458) than pork (β = −0.099) and beef (β = −0.045). This is related to the fact that meat with a higher percentage of PUFA is more prone to lipid oxidation. The addition of plant extracts influenced the rate of oxidative changes in fatty acid composition, as can be observed based on the results from Table 4.

For chicken meat, the increase in SFA share in the samples with plant extracts addition was significantly slower (β from 0.078 for thyme to 0.303 for clove) comparing to the SFA increase in the control sample (β = 0.747), and at the same time, slower decrease in PUFA in the treated samples (β from −0.094 for thyme to −0.193 for allspice) than in control (β = −0.458) was observed. MUFA share in total fatty acids increased with time of storage (apart from control, bay leaf-, and caraway-treated chicken meat) which was reflected by the positive regression coefficients, and statistically significant differences between control and samples with plant extracts were reported, with the exception of samples with basil and cardamom.

In pork meat samples, significantly higher rates of SFA changes when compared to control (β = 0.145) were reported for the samples with allspice, basil, bay leaf, black seed, cardamom, caraway, and clove (β = 0.296–0.502). For the rest of the samples, regression coefficients calculated for SFA were not significantly different from the control. Although PUFA decreased with time in all samples, a significantly faster decrease in comparison to the control was noticed for basil-treated pork (β = −0.240). The regression coefficient of pork meat with clove was −0.285, even though it was not different from the control coefficient due to high fatty acid fluctuation with time and thus lower determination coefficient (R^2^ = 0.55) and significance (*p* = 0.025) of the regression model for clove-added pork, when comparing to the model for basil-added sample (R^2^ = 0.96, *p* = 0.000). MUFA contribution in the control sample decreased during storage (β = −0.128). A similar trend was observed for allspice, bay leaf, cardamom, garlic, nutmeg, and rosemary samples. Other samples showed an increase in MUFA share with time (β = 0.081–0.225). Moreover, for allspice, caraway, and clove, no significant differences from control or not significant regression models were reported, which was the result of an increase in MUFA at the beginning of storage and then further decrease.

In the beef sample with cardamom addition, significantly faster SFA changes were noted, whereas, in the garlic sample, significantly slower SFA changes were reported in comparison to the control sample. None of the other regression coefficients calculated for SFA differed significantly from the control sample. The rate of PUFA changes was significantly faster in the black-seed-treated sample (β = −0.094, *p* = 0.011) than in control (β = −0.045). In the case of bay leaf, the regression coefficient for the PUFA index was also significantly different from the control (for the bay leaf, β = 0.027, *p* = 0.048). When allspice, basil, and garlic were added to the beef, the decrease in MUFA share was slower (significantly lower absolute values of regression coefficients) than in the control sample, whereas after the addition of clove and nutmeg, an increase with time was observed. However, in clove- and nutmeg-treated samples, MUFA increased slightly at the beginning of the storage period (up to 7 and 5 days, respectively), and then the contribution of MUFA decreased. None of the other samples showed significantly different rates of MUFA changes from the control, but the overall trend was rather downward for almost all the treated samples.

## 3. Discussion

In this study, the changes of the fatty acid composition of raw chicken, pork, and beef meat samples with the addition of plant extracts prepared from 13 commonly used spices and herbs: allspice, basil, bay leaf, black seed, cardamom, caraway, cloves, garlic, nutmeg, onion oregano, rosemary, and thyme during 13 days of storage at 4 °C were determined and analyzed.

The highest contribution of MUFA was observed in all tested meat types, with the total mean values equaling 45.8% in chicken legs and 48.5% in pork and beef neck. The predominant fatty acid was oleic acid (C18:1), with the contribution equaling 39.3, 44.6, and 45.7% in chicken, pork, and beef, respectively. Similar results were obtained by others, as chicken thigh meat was previously reported to include 43.1–49.1% of MUFA [22,23] with oleic acid at the level of 34.8% [23], the share of MUFA in pork ham was at the level of 47.9% with the 44.1% share of oleic acid [8], and in beef, MUFA share was 46.9% including oleic acid at 42% [12]. In this study, similarly to the results of others [7,8,12,22,23], PUFA contribution in total fatty acids was the highest in chicken meat and the lowest in beef meat, which resulted in the high ratio of PUFA/SFA in chicken (from 0.65 to 0.87) and low ratios PUFA/SFA in pork (from 0.19 to 0.30) and beef (from 0.06 to 0.12).

To detect some patterns in the dataset, PCA was performed as a first step of data analysis. This enabled us to show the effect of plant extract addition on the fatty acid composition during storage and detect relationships between fatty acids and the sample (source of natural antioxidants). In all meat types, two datasets were extracted. The first dataset included meat samples with allspice, bay leaf, black seed, cardamom, clove, caraway, and nutmeg. Generally, in chicken and pork meat, the highest shares of MUFA and the lowest share of PUFA were observed for these samples in comparison to control and other samples with plant extracts (basil, garlic, onion, oregano, rosemary, and thyme). In beef meat, this group of aromatic spices showed different patterns as the highest contributions of MUFA as well as PUFA were observed when comparing to control and other samples. The observed protective effect of the extracts in beef meat was in accordance with the previous report as when introducing the plant extracts to the beef meat, the most pronounced decrease in lipid oxidation expressed as TBARS values was determined for bay leaf, allspice, clove, nutmeg, caraway, and black seed [24]. Similarly, clove was shown to be the most antioxidant active extract in the lipid fraction of chicken [25] and pork [26] meat.

The second dataset extracted from the PCA included herbs and spices like basil, oregano, thyme, rosemary, garlic, and onion. The addition of these plant extracts to chicken and pork meat resulted in a higher share of PUFA in comparison to the corresponding control sample. In the beef meat samples with these extracts, PUFA and MUFA contributions were the lowest, and SFA share was the highest among all tested samples.

This could suggest that the difference between the two datasets might be in the mechanism of antioxidant action. Generally, as it was shown previously, the spices belonging to the first dataset showed very high (clove, allspice, and bay leaf) or low (black seed, cardamom, caraway, and nutmeg) antioxidant activity [25,26,27] measured as radical scavenging capacity as well as metal chelating ability whereas plant extracts from the second dataset were characterized rather by the moderate antioxidant activity [27]. Antioxidant compounds that can contribute to the antioxidant effect in meat samples include phenolic acids, flavonoids, and terpenes. They act predominantly as primary antioxidants via the mechanism of hydrogen atom donation and further delocalization of an electron in the phenol ring. However, they could also act as secondary antioxidants by chelation of transition metal ions [19]. Since beef meat contains a high amount of myoglobin and iron ions which could influence the rate of lipid oxidation, it could be suspected that allspice, bay leaf, clove, black seed, cardamom, caraway, and nutmeg in the beef meat matrix have played a role of metal chelators rather than radical scavengers and thus they showed a high protective effect against UFA oxidation in red meat which was no longer observed in such extent in chicken and pork meat. Basil, oregano, rosemary, thyme, garlic, and onion could efficiently increase the oxidative stability of PUFA in chicken and pork, acting mainly as radical scavengers. On the other hand, the concentration and qualitative characteristic of active antioxidant compounds in plant extracts, as well as the unsaturation level of fatty acids and the interaction between meat components (like lipids and proteins), could affect the final activity of spices and herbs in the meat matrix. Meat with a higher proportion of unsaturated fatty acids is more prone to the oxidation process [8]. To put more insight into this effect, further studies are needed.

The significant effect of plant extracts on fatty acid composition in various meat types during storage was shown by others. The effect could be observed after indirect dietary incorporation to meat as well as a direct addition to meat during the technological process. Dietary oregano essential oil improved oxidative stability of PUFA in pork meat [28]. A similar positive effect on the fatty acid profile was shown by Yagoubi et al. [29]. The authors proved that feeding animals with rosemary by-products maintained the unsaturation level of fatty acids in lamb meat, which resulted in a higher share of PUFA in comparison to the control sample. Kumar et al. [30] reported that black seed supplementation affected the fatty acid composition of chicken breast muscles by increasing the share of linolenic (C18:3), eicosenoic (C20:1) acids, and total PUFA. The increase was dose-dependent. Others reported that dietary black seed resulted in a remarkable increase in the chicken meat share of UFA for about 3.35% compared to control [9]. Garlic addition to raw pork minced meat significantly increased the content of linoleic acid and protected fatty acids from oxidation [31]. However, when adding thyme, rosemary, clove, and garlic to lamb burgers, no significant differences were reported in fatty acid profiles between treated samples [32].

The effect of time on fatty acid composition was statistically significant in this study. Generally, for SFA and PUFA shares, linear changes with time were reported (increase in SFA and decrease in PUFA), whereas for MUFA, the trend was upward or downward depending on the extract added, and even non-linear changes in MUFA shares were observed for some samples. The significant effect of time on fatty acid changes in meat was also observed by others. Kaczmarek et al. [31] found that the content of fatty acids in pork meat decreased linearly during refrigerated storage, and the rate of this decrease was the lowest in garlic treated pork meat comparing to control and onion- and marjoram-treated samples. Similarly to our results, an increasing proportion of SFA was observed in pork belly rib and pork loin samples during frozen storage, and at the same time, a significant decrease in the contribution of PUFA was reported in belly rib and a decrease in the share of MUFA in loin samples [8]. SFA and UFA shares also changed during chilled and frozen storage in opposite directions, meaning SFA increased and UFA decreased in beef loin [33], which is in agreement with our results. Orkusz et al. [17] reported a similar trend as it was observed in this study for chicken meat samples, i.e., SFA share in poultry meat (goose) stored at 1 °C for 14 days increased, and PUFA contribution decreased. However, no significant effect of time on the fatty acid composition of chicken breast meat was observed by Chmiel et al. [14].

Consumers expect innovations in the food industry leading to the decline of so-called “artificial” foods as they are growing in their distrust for using chemically synthesized antioxidants. Therefore, the “natural” and “clean label” claims are recently widespread marketing catchwords for food products with the addition of naturally occurring functional components from herbs and spices instead of synthetic derivatives. Herbs and spices have been widely used for ages to maintain the quality of meat during storage. According to the best knowledge of the authors, the comparison of the fatty acid composition of various meat samples with plant extract addition during refrigerated storage was set in such experimental model (13 plant extracts and three types of meat) for the first time.

In summary, as it could be observed from this study, fatty acid composition of intramuscular fat varied during storage, and the addition of plant extract significantly affected the rate of these changes. Thus, the final fatty acid composition is the result of many factors (meat matrix, time, and source of antioxidants), and the simple relation between the antioxidant activity of plant extracts in vitro and their antioxidant activity in the meat matrix is not always clear.

## 4. Materials and Methods

### 4.1. Materials

Dried allspice, basil, bay leaf, black seed, cardamom, caraway, cloves, garlic, nutmeg, onion, oregano, rosemary, and thyme were purchased from a local distributor of herbs and spices (Ciecierzyn, Poland). Chicken legs, pork neck, and beef neck were purchased from a local producer (Swarzędz, Poland). All meat cuts were minced through a 5 mm -diameter plate. Then, meat cuts were immediately transported to the laboratory. The temperature of meat during the transport was kept at 4 °C ± 2 °C.

### 4.2. Plant Extract Preparation and Characterization

The aqueous ethanol (1:1 *v*/*v*) extracts of spices and herbs were prepared according to the procedure described previously [25]. Briefly, the spices and herbs were grounded, and 15 g of each plant material was extracted with 225 mL of 50% ethanol in water in a closed container for 24 h on the magnetic stirrer in the dark. The time of extraction and ethanol concentration was chosen based on the previous study [27] to maximize the antioxidant activity and phenolic content of the extracts. Then, all extracts were filtered through 3HW Filtrak filter paper (Filtrak, Niederschlag Bärenstein, Germany) and freeze-dried.

All extracts were characterized according to their antioxidant activity and total phenolic content, and the results were shown and discussed in the previous papers [24,25,26,27].

### 4.3. Meat Sample Preparation and Storage

All meat samples with spice and herb extracts were prepared as described by Muzolf-Panek et al. [26]. Briefly, each extract was mixed separately with ground meat (chicken, pork, or beef) after dissolving the extract in water (60 mL). The final concentration of the spice/herb in meat was 0.5% (mass of spice or herb/mass of meat). Thirteen treated samples, namely with allspice, basil, bay leaf, black seed, cardamom, caraway, cloves, garlic, nutmeg, onion, oregano, rosemary, and thyme and control sample (meat without extract, only mixed with 60 mL of water) were prepared. Each sample was prepared in triplicate. Then, each meat sample with the extract (100 g) was packed in a low-density polyethylene bag and was stored at 4 °C for 13 days. Fat from each sample was extracted after 0, 3, 5, 7, and 12 days of storage as described below.

### 4.4. Fatty Acid Profiles

Fat was extracted from meat samples using chloroform–methanol mixture (2:1, *v*/*v*) according to the procedure of Folch et al. [34]. Briefly, after removing the upper phase (methanolic), the chloroform was evaporated from the samples under the nitrogen stream. All samples were stored in the freezer at −20 °C until the GC analysis. Fatty acid methyl esters were prepared according to the AOCS Official Method Ce 2-66 (1997). The fatty acid separation was performed using HP 5890 series II gas chromatographer (Hewlett Packard, Oalo Alto, USA) coupled to the flame-ionization detector and Supelcowax 10 capillary column (30 m × 0.2 mm × 0.2 mm). Operating conditions were as followed: hydrogen at a flow rate of 1.5 mL/min was used as the carrier gas, the temperature of both the injector and detector was set at 240 °C, and the oven temperature was programed from 60 °C, at 12 °C/min to 200 °C held for 25 min. Separated fatty acids were identified by comparing retention time with standard solutions. Fatty acid profiles were estimated from the chromatogram peak areas and were expressed as% of total fatty acid methyl ester (FAME) derivatives. Analysis was performed in triplicate.

### 4.5. Statistical Analysis

All statistical calculations were performed using Statistica 13.3 software (StatSoft, Tulsa, OK, USA). All experiments were set in triplicate.

PCA analysis was performed to detect patterns in data and to visualize the relationships between the fatty acid profiles and the treated samples. Before analysis, data were standardized. The fatty acid shares during the whole storage period were taken as variables. Principal components with an eigenvalue greater than 1 were extracted.

The general regression models (GRMs), namely analysis of covariance (ANCOVA) and GLM for separate slope design, were used to assess the influence of plant extracts (categorical predictor) and storage time (continuous predictor) on the fatty acid content of raw chicken, pork, and beef meat. The ANCOVA refers to experimental designs in which the first-order effect of a continuous variable (in this study time) was taken into account when evaluating the effect of categorical variable (in this study, plant extract addition). The traditional ANCOVA was applied when the continuous and categorical predictors do not interact in influencing responses on the outcome (slopes have been homogenous). When the interaction between categorical and continuous predictors was statistically significant (lack of slope homogeneity), a separate slope design was used. In a separate slope design, the effect of the continuous variable is nested within the levels of the categorical predictor variable; thus, the main effect of the continuous predictor is omitted, which is similar in form to the nested analysis of variance. Post-hoc analysis was performed using Tukey’s test. Significant differences between samples were shown at *p* ≤ 0.05. 

To compare the rates (slope of regression equation) of total fatty acid changes (SFA, MUFA, and PUFA) within the storage period between control and meat samples with different plant extracts, an MLR analysis was performed. Generally, fatty acid content increased or decreased linearly with time. The general model of MLR has the following equation:

(1) $$y=\beta_{0}+\beta_{1}t+\beta_{2}D+\beta_{3}Dt+\varepsilon$$

where: *y*—variable value (fatty acid content); *β*_0_—intercept for control; *β*_1_—regression coefficient for control sample; *β*_2–3_—regression coefficient for dummy variables; *t*—time; *D*—first dummy variable (0 or 1); *Dt*—second dummy variable (product of the first dummy variable and time); and *ε*—standard estimation error. For each sample and fatty acid, the comparisons between the coefficients of control and treated sample were performed, introducing two dummy variables as predictors to regression analysis. The first dummy variable was coded as “0” for control and “1” for the treated sample and the second dummy variable was the product of the first dummy variable and time. Since samples with plant extracts were compared to the corresponding control sample, the control sample was coded as 0, and the sample with plant extracts as 1 and then Equation (1) was as followed:

(2) $$when\;D=0,\;y=\beta_{0}+\beta_{1}t+\varepsilon ,$$

(3) $$when\;D=1,\;y=(\beta_{0}+\beta_{2})+(\beta_{1}+\beta_{3})t+\varepsilon ,$$

T-test was used to evaluate the significance of the regression coefficients for dummy variables (*p* ≤ 0.05).

## Figures and Tables

**Table 1:** Fatty acid composition (%) of raw chicken legs during 13 days of storage at 4 °C.

Spice/Herb	C14:0	C16:0	C16:1 *	C18:0	C18:1	C18:2	C18:3	C20:0	C20:1 *	SFA	MUFA	PUFA
Control	1.72 ^b^	21.42 ^e^	5.88 ^cd^	4.89 ^de^	39.59 ^def^	20.49 ^c^	2.00 ^e^	0.14 ^b^	0.33 ^ab^	30.40 ^de^	46.15 ^d^	23.29 ^de^
Allspice	2.59 ^e^	20.86 ^cd^	6.68 ^f^	4.61 ^cd^	39.80 ^ef^	18.42 ^a^	1.68 ^a^	0.01 ^a^	0.49 ^bc^	31.58 ^g^	47.59 ^e^	20.66 ^a^
Basil	0.95 ^a^	21.34 ^e^	5.69 ^bc^	5.20 ^ef^	40.00 ^ef^	22.17 ^ef^	2.15 ^f^	0.05 ^ab^	0.31 ^a^	28.49 ^a^	46.19 ^d^	25.25 ^fg^
Bay leaf	2.06 ^c^	21.01 ^d^	6.44 ^def^	3.13 ^a^	40.09 ^f^	21.09 ^d^	1.82 ^bc^	0.01 ^a^	0.42 ^abc^	28.96 ^ab^	47.41 ^e^	23.47 ^e^
Black seed	2.22 ^cd^	20.60 ^abc^	6.32 ^c–f^	4.84 ^d^	38.83 ^b^	20.64 ^cd^	1.75 ^ab^	0.01 ^a^	0.52 ^c^	30.55 ^e^	46.24 ^d^	23.04 ^de^
Cardamom	2.29 ^d^	20.82 ^cd^	5.99 ^cde^	5.34 ^fg^	38.21 ^a^	20.41 ^c^	1.89 ^d^	0.02 ^a^	0.48 ^bc^	31.45 ^fg^	45.22 ^c^	22.98 ^de^
Clove	2.41 ^de^	20.30 ^a^	6.73 ^f^	4.46 ^c^	38.65 ^ab^	20.43 ^c^	1.83 ^cd^	0.04 ^a^	0.48 ^bc^	30.52 ^de^	46.43 ^d^	22.90 ^cd^
Caraway	2.31 ^d^	20.70 ^bcd^	6.59 ^ef^	4.12 ^b^	39.83 ^ef^	19.37 ^b^	1.68 ^a^	0.09 ^ab^	0.45 ^abc^	30.49 ^de^	47.46 ^e^	21.69 ^b^
Garlic	1.03 ^a^	21.80 ^f^	5.11 ^b^	5.52 ^gh^	39.49 ^cde^	22.02 ^ef^	2.22 ^fg^	0.07 ^ab^	0.34 ^ab^	29.45 ^bc^	45.14 ^bc^	25.33 ^fg^
Nutmeg	2.36 ^d^	20.48 ^ab^	6.97 ^f^	3.91 ^b^	39.77 ^ef^	20.17 ^c^	1.71 ^a^	0.01 ^a^	0.39 ^abc^	29.70 ^bcd^	47.66 ^e^	22.44 ^c^
Onion	1.12 ^a^	22.26 ^h^	4.37 ^a^	5.99 ^ij^	39.10 ^bcd^	21.83 ^e^	2.24 ^gh^	0.06 ^ab^	0.40 ^abc^	30.62 ^ef^	44.14 ^a^	25.17 ^f^
Oregano	1.05 ^a^	22.01 ^fgh^	4.23 ^a^	6.11 ^j^	39.12 ^bcd^	22.14 ^ef^	2.24 ^gh^	0.08 ^ab^	0.40 ^abc^	30.34 ^de^	43.99 ^a^	25.67 ^fg^
Rosemary	1.11 ^a^	21.89 ^fg^	4.46 ^a^	5.71 ^hi^	39.19 ^bcd^	22.35 ^f^	2.35 ^i^	0.07 ^ab^	0.35 ^ab^	29.93 ^cde^	44.22 ^ab^	25.78 ^g^
Thyme	1.10 ^a^	22.17 ^gh^	4.12 ^a^	6.01 ^ij^	38.96 ^bc^	22.24 ^ef^	2.29 ^hi^	0.07 ^ab^	0.36 ^ab^	30.52 ^de^	43.70 ^a^	25.66 ^fg^

Mean values were calculated for the levels of categorical predictor variable and include the effect of time (total mean values); * significant homogeneity of slopes; ^a–j^ values with the identical upper letter index (in the column) do not differ significantly (*p* > 0.05, Tukey’s test).

**Table 2:** Fatty acid composition (%) of raw pork neck during 13 days of storage at 4 °C.

Spice/Herb	C14:0 *	C16:0	C16:1	C18:0	C18:1	C18:2	C18:3	C20:0	C20:1	SFA	MUFA	PUFA
Control	1.43 ^b^	25.14 ^abc^	3.16 ^b^	14.46 ^de^	45.05 ^cd^	8.54 ^abc^	0.60 ^efg^	0.12 ^abc^	0.78 ^b^	41.15 ^abc^	48.99 ^def^	9.14 ^abc^
Allspice	1.68 ^c^	25.30 ^bc^	4.68 ^d^	13.11 ^a^	45.28 ^d^	7.80 ^a^	0.54 ^b–e^	0.10 ^ab^	0.92 ^bc^	40.20 ^a^	50.88 ^g^	8.35 ^a^
Basil	1.22 ^a^	24.46 ^a^	2.21 ^a^	14.75 ^ef^	44.77 ^cd^	10.28 ^d^	0.56 ^cde^	0.09 ^a^	0.50 ^a^	40.52 ^a^	47.48 ^bc^	10.84 ^d^
Bay leaf	1.66 ^c^	25.83 ^cd^	3.69 ^bc^	14.58 ^de^	44.55 ^cd^	7.41 ^a^	0.64 ^fg^	0.13 ^bc^	0.97 ^c^	42.19 ^cd^	49.21 ^ef^	8.05 ^a^
Black seed	1.65 ^c^	26.13 ^d^	3.53 ^bc^	14.23 ^cd^	44.28 ^bc^	7.96 ^a^	0.59 ^def^	0.15 ^c^	0.92 ^bc^	42.16 ^cd^	48.73 ^c–f^	8.54 ^a^
Cardamom	1.66 ^c^	25.71 ^cd^	3.72 ^bc^	13.51 ^ab^	45.28 ^d^	8.00 ^a^	0.53 ^bcd^	0.09 ^a^	0.87 ^bc^	40.97 ^ab^	49.86 ^efg^	8.53 ^a^
Clove	1.67 ^c^	25.48 ^cd^	4.03 ^cd^	13.80 ^bc^	44.56 ^cd^	8.34 ^ab^	0.54 ^bcd^	0.11 ^abc^	0.89 ^bc^	41.06 ^abc^	49.48 ^efg^	8.88 ^ab^
Caraway	1.63 ^c^	25.83 ^cd^	3.95 ^bcd^	15.14 ^f^	43.63 ^ab^	7.65 ^a^	0.54 ^b–e^	0.09 ^a^	0.92 ^bc^	42.69 ^d^	48.50 ^cde^	8.19 ^a^
Garlic	1.31 ^ab^	25.88 ^cd^	2.18 ^a^	14.46 ^de^	44.99 ^cd^	9.35 ^bcd^	0.50 ^abc^	0.09 ^ab^	0.40 ^a^	41.74 ^bcd^	47.57 ^bcd^	9.85 ^bcd^
Nutmeg	1.88 ^d^	25.40 ^bcd^	4.20 ^cd^	13.65 ^b^	44.89 ^cd^	7.77 ^a^	0.59 ^def^	0.11 ^ab^	0.94 ^bc^	41.02 ^abc^	50.03 ^fg^	8.36 ^a^
Onion	1.32 ^ab^	25.86 ^cd^	2.07 ^a^	14.57 ^de^	45.00 ^cd^	9.44 ^bcd^	0.45 ^a^	0.11 ^ab^	0.37 ^a^	41.86 ^bcd^	47.43 ^bc^	9.89 ^bcd^
Oregano	1.31 ^ab^	25.87 ^cd^	2.25 ^a^	14.85 ^ef^	44.41 ^bcd^	9.40 ^bcd^	0.49 ^ab^	0.10 ^ab^	0.42 ^a^	42.13 ^bcd^	47.07 ^ab^	9.89 ^bcd^
Rosemary	1.25 ^a^	24.59 ^ab^	2.14 ^a^	14.57 ^de^	43.15 ^a^	11.60 ^e^	0.66 ^g^	0.10 ^ab^	0.52 ^a^	40.51 ^a^	45.80 ^a^	12.27 ^e^
Thyme	1.30 ^ab^	25.34 ^bcd^	2.26 ^a^	14.40 ^de^	44.96 ^cd^	9.73 ^cd^	0.56 ^b–e^	0.11 ^ab^	0.45 ^a^	41.14 ^abc^	47.66 ^bcd^	10.28 ^cd^

Mean values were calculated for the levels of categorical predictor variable and include the effect of time (total mean values); * significant homogeneity of slopes; ^a–g^, values with the identical upper letter index (in column) do not differ significantly (*p* > 0.05, Tukey’s test).

**Table 3:** Fatty acid composition (%) of raw beef neck during 13 days of storage at 4 °C.

Spice/Herb	C14:0	C16:0 *	C16:1	C18:0	C18:1	C18:2	C18:3	C20:0	C20:1 *	SFA *	MUFA *	PUFA
Control	2.11 ^e^	26.55 ^e^	2.22 ^a^	17.87 ^d^	45.87 ^c^	3.05 ^bf^	0.43 ^c^	0.07 ^a^	0.18 ^a^	47.38 ^c^	48.27 ^cd^	3.73 ^b^
Allspice	1.89 ^bc^	25.67 ^cd^	2.36 ^ab^	17.17 ^c^	48.00 ^ef^	3.17 ^bcf^	0.56 ^de^	0.08 ^a^	0.16 ^a^	45.69 ^b^	50.52 ^fg^	4.25 ^c^
Basil	2.26 ^f^	26.82 ^e^	2.99 ^c^	20.04 ^gh^	44.10 ^b^	2.35 ^a^	0.20 ^a^	0.08 ^a^	0.18 ^a^	49.96 ^de^	47.27 ^abc^	2.80 ^a^
Bay leaf	1.95 ^d^	25.64 ^cd^	2.35 ^ab^	17.21 ^c^	46.92 ^cde^	3.84 ^d^	0.62 ^fg^	0.08 ^a^	0.18 ^a^	45.58 ^b^	49.45 ^def^	4.69 ^de^
Black seed	1.72 ^a^	25.19 ^ab^	2.34 ^ab^	16.60 ^b^	47.99 ^ef^	4.56 ^e^	0.51 ^d^	0.08 ^a^	0.17 ^a^	44.39 ^a^	50.49 ^fg^	5.51 ^f^
Cardamom	1.91 ^cd^	26.00 ^d^	2.23 ^a^	18.16 ^d^	46.71 ^cd^	3.20 ^bc^	0.58 ^ef^	0.08 ^a^	0.15 ^a^	46.95 ^c^	49.09 ^de^	4.37 ^cd^
Clove	1.85 ^b^	25.05 ^a^	2.19 ^a^	16.65 ^b^	47.15 ^def^	3.96 ^d^	0.65 ^g^	0.08 ^a^	0.22 ^a^	44.45 ^a^	49.56 ^ef^	4.92 ^e^
Caraway	1.95 ^cd^	25.55 ^bc^	2.22 ^a^	17.36 ^c^	47.56 ^def^	3.43 ^c^	0.60 ^efg^	0.08 ^a^	0.16 ^a^	45.73 ^b^	49.94 ^efg^	4.33 ^cd^
Garlic	2.56 ^i^	27.59 ^f^	3.19 ^d^	19.22 ^f^	43.38 ^ab^	2.38 ^a^	0.28 ^b^	0.08 ^a^	0.18 ^a^	50.26 ^e^	46.75 ^ab^	2.88 ^a^
Nutmeg	2.21 ^f^	25.94 ^cd^	2.49 ^b^	16.10 ^a^	48.13 ^f^	3.16 ^bc^	0.71 ^i^	0.08 ^a^	0.18 ^a^	45.07 ^ab^	50.80 ^g^	4.05 ^bc^
Onion	2.46 ^h^	27.31 ^f^	3.07 ^cd^	19.68 ^g^	43.46 ^ab^	2.43 ^a^	0.25 ^ab^	0.09 ^a^	0.18 ^a^	50.32 ^e^	46.71 ^ab^	2.89 ^a^
Oregano	2.36 ^g^	27.37 ^f^	3.17 ^cd^	18.81 ^e^	44.05 ^b^	2.59 ^af^	0.27 ^b^	0.08 ^a^	0.18 ^a^	49.40 ^d^	47.39 ^bc^	3.12 ^a^
Rosemary	2.48 ^h^	27.63 ^f^	3.17 ^cd^	19.21 ^f^	43.45 ^ab^	2.42 ^af^	0.27 ^b^	0.08 ^a^	0.18 ^a^	50.20 ^e^	46.79 ^ab^	2.93 ^a^
Thyme	2.42 ^h^	27.38 ^f^	3.04 ^cd^	20.35 ^h^	42.87 ^a^	2.33 ^af^	0.25 ^ab^	0.09 ^a^	0.18 ^a^	51.02 ^f^	46.08 ^a^	2.79 ^a^

Mean values were calculated for the levels of categorical predictor variable and include the effect of time (total mean values); * significant homogeneity of slopes; ^a–i^, values with the identical upper letter index (in column) do not differ significantly (*p* > 0.05, Tukey’s test).

**Table 4:** The values of regression coefficients (β) determined by the multiple linear regression analysis (MLR) for saturated fatty acid (SFA), monounsaturated fatty acid (MUFA), and polyunsaturated fatty acid (PUFA) changes of raw chicken, pork, and beef samples during refrigerated storage at 4 °C.

	Allspice	Basil	Bay Leaf	Black Seed	Caraway	Cardamom	Clove	Garlic	Nutmeg	Onion	Oregano	Rosemary	Thyme	Control
**Chicken**														
SFA	0.182 *	0.216 *	0.189 *	0.161 *	0.235 *	0.184 *	0.303 *	0.148 *	0.120 *	0.100 *	0.153 *	0.111 *	0.078 *	0.747
MUFA	0.038 *	0.167 ^N^	−0.012 *	0.056 *	−0.096	0.038	0.120 *^N^	0.115 *	0.053 *	0.076 *	0.053 *	0.117 *	0.026 *	−0.065
PUFA	−0.193 *	−0.093 *	−0.080 *	−0.123 *	−0.126 *	−0.115 *	−0.171 *	−0.100 *	−0.055 *	−0.085 *	−0.128 *	−0.130 *	−0.094 *	−0.458
**Pork**														
SFA	0.502 *	0.296 *	0.364 *	0.367 *	0.431 *	0.439 *	0.399 *	0.188	0.239	0.195	0.146	0.147	0.178	0.145
MUFA	−0.309	0.225 *	−0.075 ^N^	0.149 *	0.151 *^N^	−0.268 *	0.146 ^N^	−0.179	−0.272 *	0.081 *	0.158 *	−0.343 *	0.120 *	−0.128
PUFA	−0.147	−0.240 *	−0.048	−0.097	−0.053	−0.071 ^N^	−0.285	−0.064	−0.058	−0.089	−0.117	−0.211	−0.063	−0.099
**Beef**														
SFA	0.178	0.165	0.179	0.213	0.200	0.259 *	0.161	0.109 *	0.167	0.203	0.222	0.134	0.212	0.171
MUFA	−0.110 *	−0.015 *	−0.190	−0.145	−0.168	−0.047 ^N^	0.082 *	−0.042 *	0.013 *	−0.091	−0.144	−0.125	−0.110	−0.172
PUFA	−0.047	−0.049	0.027 *	−0.094 *	−0.037	−0.080	−0.072	−0.037	−0.027	−0.044	−0.068	−0.023	−0.037	−0.045

* indicated significantly different regression coefficients from control sample (*p* ≤ 0.05); ^N^ indicated not statistically significant regression model (*p* > 0.05).

## Data Availability

The data presented in this study is available upon reasonable request.

## Supplementary Materials

The following are available online. Table S1: Fatty acid composition (%) in various meat samples stored at 4 °C.
