# Supplementary material for: Chemometric Analysis of Fatty Acid Composition of Raw Chicken, Beef, and Pork Meat with Plant Extract Addition during Refrigerated Storage
Source: Molecules. 2021 Aug 16;26(16):4952. doi: 10.3390/molecules26164952 (PMC8399115; doi:10.3390/molecules26164952)
Supplement: Supplementary file 1 [file molecules-26-04952-s001.zip › molecules-1272416-supplementary.pdf]

Table S1. Fatty acid composition (%) in various meat samples stored at 4°C.

plant extract	day of storage	chicken			pork			beef		
		SFA	MUFA	PUFA	SFA	MUFA	PUFA	SFA	MUFA	PUFA
control	0	26.53	46.51	25.39	40.27	50.025	10.02	46.44	49.21	4.12
	3	27.48	46.46	25.19	40.67	49.22	9.35	46.69	48.97	3.77
	5	27.93	46.31	24.99	40.86	48.85	9.01	47.14	48.74	3.74
	7	32.49	45.93	22.34	41.39	48.77	8.96	47.57	47.82	3.69
	10	33.26	45.84	21.28	41.78	49.14	8.79	47.99	47.46	3.54
	12	34.71	45.86	20.59	41.91	47.96	8.73	48.42	47.43	3.53
allspice	0	30.72	47.51	21.56	38.29	51.51	9.25	44.78	50.94	4.60
	3	31.1	47.26	21.36	38.51	52.39	8.80	45.09	50.87	4.32
	5	31.25	47.61	20.97	38.77	51.74	8.65	45.45	50.83	4.33
	7	31.34	47.49	20.69	39.13	52.07	8.11	45.63	50.66	4.16
	10	31.61	47.83	20.40	42.10	49.63	7.66	45.96	50.38	4.06
	12	33.45	47.83	18.95	44.39	47.95	7.60	47.20	49.42	4.02
basil	0	27.24	44.77	25.87	38.72	46.50	12.13	49.08	47.08	3.09
	3	27.70	46.64	25.62	39.47	46.72	11.66	49.29	47.50	3.06
	5	28.16	46.16	25.22	40.23	46.63	11.52	49.69	47.30	2.79
	7	28.76	45.34	25.09	40.81	47.38	10.51	50.07	47.34	2.72
	10	29.40	46.56	24.99	41.70	48.83	9.70	50.80	47.88	2.61
	12	29.70	47.64	24.72	42.19	48.79	9.51	50.84	46.53	2.55
bay leaf	0	27.66	47.39	23.95	39.71	49.56	8.29	45.18	50.39	4.75
	3	28.68	47.71	23.70	41.47	49.21	8.21	44.90	50.25	4.26
	5	28.79	47.27	23.55	41.97	48.84	8.20	44.23	49.53	4.48
	7	28.93	47.41	23.49	42.33	50.51	8.03	45.67	49.52	5.07
	10	29.24	47.30	23.20	42.72	48.79	7.86	46.65	49.16	4.68
	12	30.43	47.39	22.94	44.93	48.33	7.72	46.83	47.85	4.88
black seed	0	29.61	45.91	23.78	40.33	47.87	9.09	43.00	51.38	6.23
	3	29.78	45.96	23.45	41.00	48.18	8.79	43.52	51.35	5.73
	5	30.46	45.92	23.14	41.25	48.33	8.73	44.44	50.34	5.49
	7	30.92	46.71	22.89	42.06	49.14	8.61	44.72	50.06	5.37
	10	31.07	46.58	22.85	43.59	49.36	8.11	45.08	50.02	5.24
	12	31.46	46.34	22.13	44.70	49.50	7.92	45.57	49.79	5.00
cardamom	0	30.19	45.04	23.71	38.35	51.82	8.89	45.39	48.46	4.79
	3	31.10	45.56	23.31	39.63	50.57	8.32	46.17	49.25	4.69
	5	31.18	44.81	23.16	40.48	50.00	8.12	46.66	49.64	4.55
	7	31.57	44.90	22.83	41.39	49.38	10.26	47.11	50.50	4.22
	10	32.11	45.04	22.59	41.69	48.84	7.91	47.56	49.10	4.11
	12	32.54	45.94	22.29	44.27	48.55	7.68	48.78	47.61	3.86
clove	0	28.36	45.63	24.23	39.13	47.18	12.02	43.53	48.13	5.25
	3	29.06	46.09	23.48	39.26	49.21	8.83	43.95	49.44	5.05
	5	31.03	45.64	22.82	40.67	50.27	8.36	44.07	50.06	5.42
	7	31.28	47.27	22.38	41.30	51.05	8.11	44.62	50.85	4.72
	10	31.47	47.42	22.27	41.77	50.58	8.07	45.22	49.61	4.60
	12	31.94	46.51	22.19	44.22	48.59	7.89	45.33	49.25	4.46

caraway	0	28.98	47.54	22.31	38.98	47.82	8.48	44.67	51.06	4.58
	3	29.86	47.53	22.17	42.02	47.61	8.44	44.96	50.35	4.42
	5	30.32	48.25	21.90	43.14	47.69	8.27	45.62	50.29	4.35
	7	30.55	48.47	21.62	43.32	49.10	8.03	45.71	49.42	4.28
	10	31.14	46.11	21.38	43.53	50.48	8.01	46.10	49.79	4.22
	12	32.06	46.86	20.73	45.16	48.31	7.89	47.32	48.74	4.10
garlic	0	28.58	44.35	25.95	40.62	48.61	10.32	49.47	47.05	3.13
	3	28.83	45.21	25.68	41.20	48.02	10.01	50.07	46.85	2.93
	5	29.41	44.97	25.37	41.54	47.76	9.87	50.18	46.80	2.90
	7	29.60	44.81	25.26	41.83	47.64	9.74	50.29	46.68	2.91
	10	29.95	45.32	25.01	42.12	47.23	9.63	50.62	46.56	2.79
	12	30.34	46.18	24.71	43.14	46.14	9.51	50.91	46.56	2.61
nutmeg	0	28.87	47.46	22.76	40.02	51.70	8.65	43.47	50.34	4.13
	3	29.31	47.28	22.62	40.29	50.53	8.59	45.07	50.66	3.91
	5	29.69	47.53	22.48	40.46	50.40	8.44	45.13	51.53	4.64
	7	29.84	47.98	22.43	40.84	50.20	8.33	45.28	50.82	3.92
	10	30.14	47.66	22.24	40.99	49.47	8.16	45.58	50.87	3.81
	12	30.34	48.07	22.08	43.54	47.88	7.96	45.86	50.56	3.89
onion	0	30.06	43.84	25.75	40.51	46.69	10.59	49.02	46.95	3.16
	3	30.25	43.80	25.36	41.16	47.16	10.02	49.71	46.95	3.16
	5	30.42	43.96	25.26	42.00	47.16	9.95	49.97	47.32	2.98
	7	30.82	43.94	25.10	42.08	48.26	9.74	50.77	46.94	2.59
	10	30.97	44.86	24.85	42.42	48.00	9.70	50.87	45.93	2.62
	12	31.22	44.44	24.69	42.98	47.33	9.36	51.56	46.15	2.82
oregano	0	29.44	43.23	26.48	41.36	45.68	10.82	48.47	47.97	3.36
	3	29.85	43.91	26.18	41.71	46.58	10.14	48.81	47.88	3.33
	5	30.23	44.51	25.76	41.89	47.29	9.88	48.78	47.71	3.58
	7	30.38	44.02	25.35	41.99	47.65	9.69	48.97	47.35	3.01
	10	30.74	44.29	25.25	42.57	47.62	9.54	49.89	47.79	2.84
	12	31.42	43.95	24.98	43.24	47.61	9.28	51.47	45.64	2.62
rosemary	0	29.36	43.71	26.49	39.72	47.35	13.37	49.60	47.72	2.91
	3	29.55	43.85	26.18	39.49	46.86	14.64	49.60	47.47	2.91
	5	29.72	44.15	26.08	40.61	46.48	11.10	50.01	46.49	3.13
	7	29.81	43.80	25.69	41.06	46.25	11.24	50.13	46.34	3.08
	10	30.54	44.44	25.34	40.79	45.19	12.05	50.76	46.50	3.10
	12	30.57	45.36	24.90	41.37	42.67	11.20	51.11	46.22	2.42
thyme	0	30.15	43.31	26.24	40.26	46.82	10.69	49.99	46.85	2.98
	3	30.28	43.76	25.92	40.48	47.23	10.52	50.05	46.85	2.98
	5	30.34	43.55	25.76	40.81	47.71	10.28	50.49	46.30	2.79
	7	30.45	44.27	25.57	41.17	47.86	10.17	51.50	44.95	2.75
	10	30.66	43.60	25.48	41.66	48.05	10.14	51.73	45.50	2.71
	12	31.21	43.72	24.97	42.46	48.29	9.89	52.35	46.04	2.52
